# Randomized control trials demonstrate that nutrition-sensitive social protection interventions increase the use of multiple-micronutrient powders and iron supplements in rural pre-school Bangladeshi children

**DOI:** 10.1017/S1368980017004232

**Published:** 2018-02-22

**Authors:** John Hoddinott, Akhter Ahmed, Shalini Roy

**Affiliations:** 1 Division of Nutrition Sciences, Cornell University, Savage Hall, Room 305, Ithaca, NY 14850, USA; 2 International Food Policy Research Institute (IFPRI), Dhaka, Bangladesh; 3 International Food Policy Research Institute (IFPRI), Washington, DC, USA

**Keywords:** Social protection, Bangladesh, Multiple-micronutrient powders, Nutrition behaviour change communication

## Abstract

**Objective:**

To examine the impact of a nutrition-sensitive social protection intervention on mothers’ knowledge of Fe deficiency, awareness of multiple-micronutrient powders (MMP) and the consumption of MMP and other Fe supplements by their children aged 6–59 months.

**Design:**

Two randomized controlled trials with treatment arms including cash transfers, food transfers, cash and food transfers, cash and nutrition behaviour change communication (BCC), and food and nutrition BCC were implemented over two years. Both included a control group that received no transfer or BCC. Transfer recipients were mothers living in poor households with at least one child aged less than 2 years at baseline. Probit models were used to analyse endline data.

**Setting:**

Rural areas in north-west and south Bangladesh.

**Subjects:**

Mothers (*n* 4840) and children 6–59 months (*n* 4840).

**Results:**

A transfer accompanied by nutrition BCC increased the share of mothers with knowledge of Fe deficiency (11·9 and 9·2 percentage points for North and South, respectively, *P*≤0·01), maternal awareness of MMP (29·0 and 22·2 percentage points, *P*≤0·01), the likelihood that their children 6–59 months had ever consumed MMP (32 and 11·9 percentage points, *P*≤0·01), consumed MMP in the preceding week (16·9 and 3·9 percentage points, *P*≤0·01) and consumed either MMP or an Fe supplement in the preceding week (22·3 and 7·1 percentage points, *P*≤0·01). Improvements were statistically significant relative to groups that received a transfer only.

**Conclusions:**

Nutrition-sensitive social protection (transfers with BCC added) may be a promising way to advance progress on micronutrient deficiencies.

Child undernutrition in all its forms is widespread in much of the developing world. Among children less than 5 years of age, 165 million are stunted, 51 million are wasted, 90 million are subclinically deficient in vitamin A and 18 % suffer from Fe-deficiency anaemia (IDA)^(^ ^1^ ^)^. Many of these forms of undernutrition have long-term consequences. For example, IDA among children of pre-school age is linked to poorer cognitive, motor and social-emotional function in adolescence^(^ ^2^ ^,^ ^3^ ^)^. Animal models point to Fe deficiency causing direct biological deficits in neurometabolism, myelination and neurotransmitter function^(^ ^2^ ^)^.

IDA in pre-school children can be addressed in a number of ways. Nutrition-specific interventions and programmes addressing IDA focus on improving diet, supplementation and fortification^(^ ^1^ ^,^ ^4^ ^)^. Within this approach, provision of free supplements such as multiple-micronutrient powders (MMP)^(^ ^5^ ^)^ – single-dose sachets containing Fe and other micronutrients in a powdered form, which can be sprinkled on to foods prepared in the household – has proved successful in reducing IDA among children of pre-school age in several developing countries including Bangladesh^(^ ^6^ ^)^, Ghana^(^ ^7^ ^)^ and Haiti^(^ ^8^ ^)^. However, nutrition-specific interventions such as free direct provision of supplements^(^ ^1^ ^)^ have limited sustainability and scalability; market-based strategies that rely on voluntary purchase and use by households are needed^(^ ^9^ ^)^. These market-based strategies rely on the availability of MMP or other supplements in pharmacies or shops; household incomes that are high enough for these supplements to be affordable; and knowledge among caregivers that these supplements will benefit their children^(^ ^9^ ^,^ ^10^ ^)^. Conditional on MMP availability, nutrition-sensitive interventions may be one means of ensuring households have sufficient income to purchase supplements^(^ ^1^ ^,^ ^10^ ^)^. Nutrition-sensitive interventions draw on complementary sectors (such as agriculture, social protection and health) to affect the underlying determinants of nutritional status, including factors underlying household decisions related to nutrition^(^ ^10^ ^)^. Social protection interventions – specifically, programmes that provide cash or in-kind transfers to poor households – are seen as particularly promising vehicles for addressing undernutrition in all its forms, as these reach large numbers of poor households which may be particularly constrained in nutrition-related decisions^(^ ^10^ ^)^. However, assessments of the impact of these interventions on IDA are limited in number^(^ ^10^ ^)^. These are dominated by one type of social protection programme, conditional cash transfers, and one region of the world, Latin America^(^ ^10^ ^,^ ^11^ ^)^. These programmes, where cash transfers were accompanied by the provision for pre-school child consumption of either micronutrient powders (Mexico) or Fe tablets (Honduras and Nicaragua), showed mixed results^(^ ^11^ ^)^. Anaemia in pre-school children was reduced in Mexico but there was no effect in either Honduras or Nicaragua^(^ ^11^ ^)^. Further, to the best of our knowledge, it has not been determined if raising incomes is sufficient to increase use of MMP or other micronutrient supplements, or if this needs to be complemented by activities that improve caregivers’ knowledge of micronutrient deficiencies and how these can be addressed.

The present paper provides new evidence on the impact of a nutrition-sensitive social protection intervention on the use of supplements that address IDA among children. We assessed whether transfers received as part of a social protection intervention are sufficient to induce use of supplements, whether the addition of a high-quality nutrition behaviour change communication (BCC) intervention has additional effects on use and whether the form of the transfer, cash or food, affects the use of these supplements. We assessed this using two cluster-randomized controlled trials in Bangladesh, varying the type of transfer provided – cash or food – with or without BCC.

## Methods

### Programme description

**Fig. 1:**
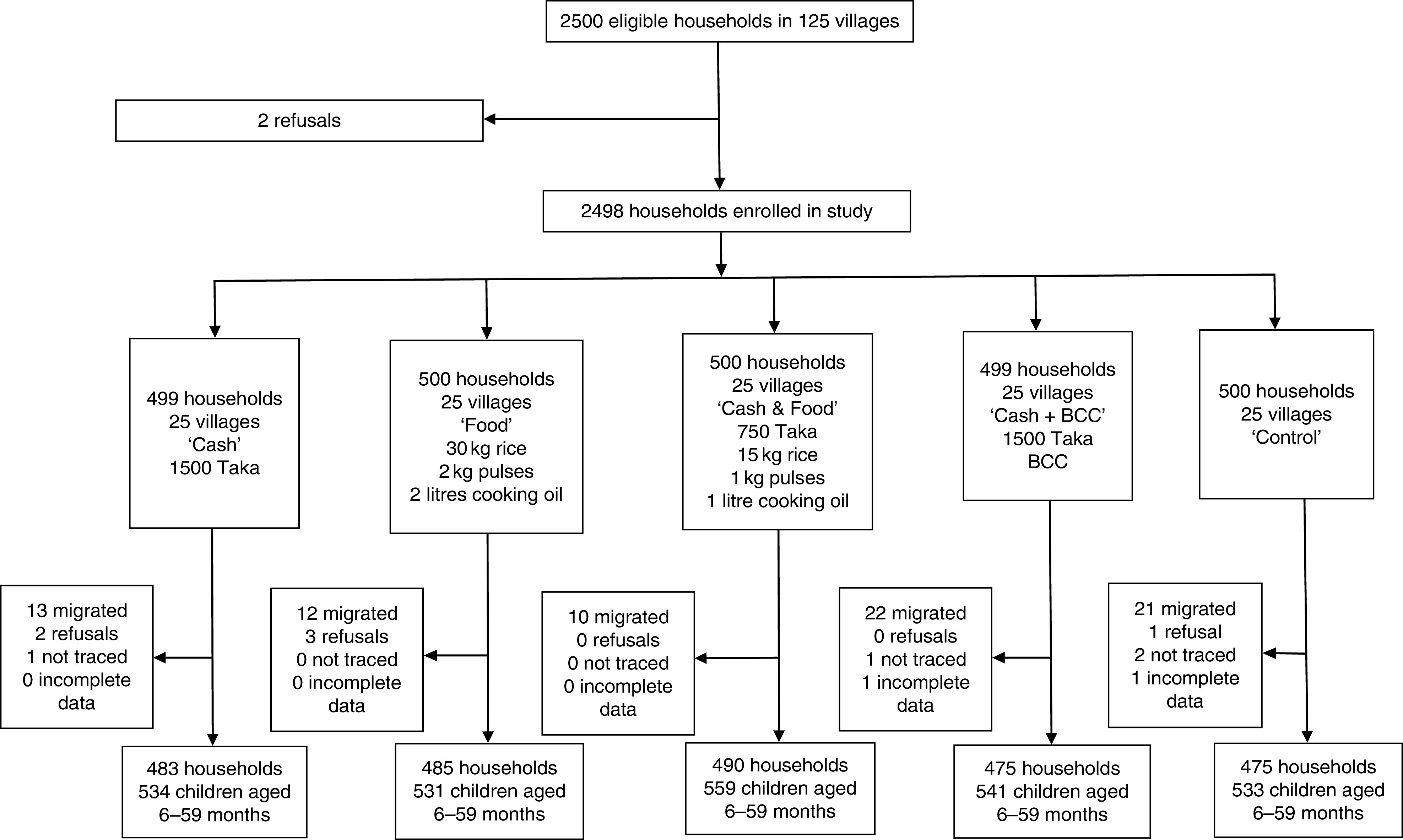
Flow diagram of participant participation in the North randomized controlled trial conducted in north-west Bangladesh, March 2012–May 2014 (BCC, (high-quality nutrition) behaviour change communication)

**Fig. 2:**
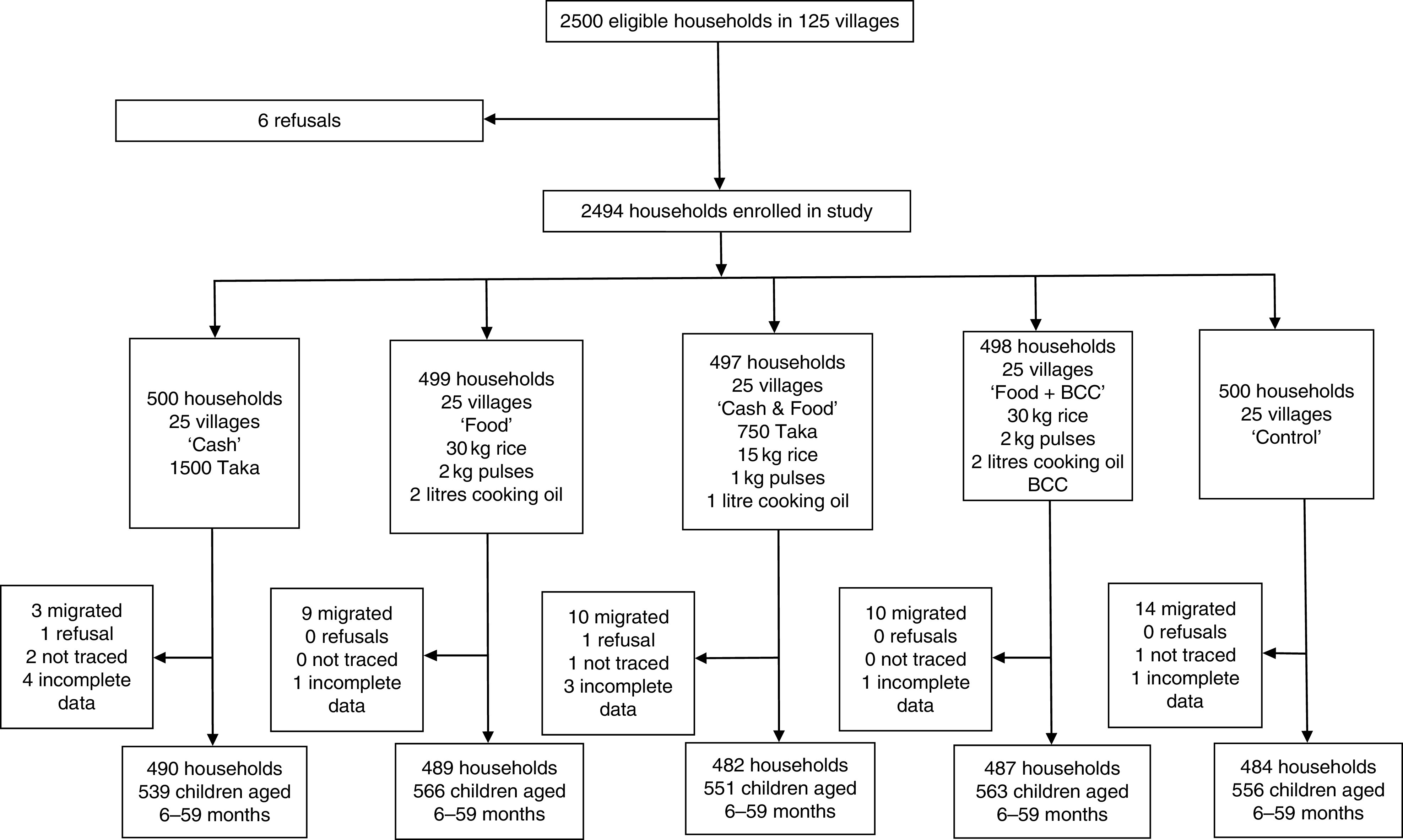
Flow diagram of participant participation in the South randomized controlled trial conducted in south Bangladesh, March 2012–May 2014 (BCC, (high-quality nutrition) behaviour change communication)

Between March 2012 and May 2014, the Transfer Modality Research Initiative (TMRI) implemented two cluster-randomized controlled trials (RCT): one in rural areas of the north-west region of Bangladesh (‘North RCT’) and one in the south (‘South RCT’)^(^ ^12^ ^)^. In the North, study villages were randomly assigned to a control group or one of four treatment arms in which beneficiaries received one of the following monthly: (i) a cash transfer of 1500 Taka (‘Cash’), approximately $US 19 and equivalent to approximately 25 % of average monthly household consumption expenditures of poor rural households in Bangladesh^(^ ^12^ ^)^; (ii) an equal-value food ration consisting of rice, lentils and micronutrient-fortified cooking oil (‘Food’); (iii) a half cash transfer and half food ration (‘Cash & Food’); or (iv) the cash transfer along with nutrition BCC (‘Cash + BCC’; see Figs 1 and 2 for further details). The control group did not receive food, cash or nutrition BCC. In the South, study villages were also randomly assigned to a control group or one of four treatment arms; the first three treatment groups were the same as in the North. The final treatment group in the South was different: beneficiaries received the food ration along with nutrition BCC (‘Food + BCC’). All transfer payments and BCC activities were implemented for 24 months. Quantities were chosen so that the value of the food ration was equal to the value of the cash provided in the cash treatment arm, as of March 2012. Payments were made to mothers who met study inclusion criteria at baseline; specifically, they lived in poor households (see ‘Sample design’ section for details) and had at least one child aged 0–24 months.

The nutrition BCC consisted of a suite of intensive activities. The core activity consisted of a weekly, one-hour group session of the ten beneficiaries in each village with a trained community nutrition worker (CNW). These sessions covered a defined series of topics: nutrition, diet diversity and health; handwashing, hygiene and health; diet diversity and micronutrients; breast-feeding; complementary foods for children aged 6–24 months; feeding and treatment of children with diarrhoea; maternal nutrition; encouraging homestead food production; and women’s status and relationships with influential family members such as husbands and mothers-in-law (e.g. negotiating intrahousehold relationships related to feeding pre-school children). Methods used to deliver this information included presentations, question and answer, interactive call and answer, songs and chants, practical demonstrations and role playing. Micronutrient deficiencies and how these could be addressed were discussed approximately once per month. CNW made home visits to beneficiaries, to follow up on topics discussed during the group sessions and to discuss specific concerns that mothers might have. While attendance at the BCC sessions was a condition for receipt of transfers in the ‘Cash + BCC’ and ‘Food + BCC’ arms, this was a soft condition. When a mother missed a session, the CNW followed up with a home visit to ascertain why the session had been missed and discuss the session’s material; no beneficiary was dropped from the study for failing to attend sessions.

MMP and Fe supplements were not directly provided as part of the BCC activities. However, the BCC emphasized the importance of micronutrients including Fe in the diets of children aged 6–59 months and encouraged use of MMP and other Fe supplements. MMP, specifically a five-ingredient micronutrient powder (branded as *Pushtikona* and containing 12·5 mg ferrous fumerate, 0·16 mg folic acid, 0·3 mg vitamin A (acetate), 30 mg vitamin C and 5 mg zinc gluconate), was available through the ‘Bangladesh Sprinkles Program’, a partnership between non-governmental organizations BRAC and the Global Alliance for Improved Nutrition (GAIN)^(^ ^13^ ^)^. At baseline, a single-dose sachet of *Pushtikona* cost 1·55 Taka^(^ ^14^ ^)^. Over a 6-month period, sixty doses are recommended^(^ ^13^ ^)^, implying that the per month cost of *Pushtikona* is 15·5 Taka or 1·03 % of the monthly cash transfer. Other Fe supplements such as tablets or syrups were also available through health clinics or pharmacies in many study areas.

### Sample design

We used a cluster-randomized controlled trial design; see Figs 1 and 2. In each region, five sub-districts (*upazilas*) were selected from a list of *upazilas* where, in 2010, the proportion of households living below the lower poverty line in Bangladesh (approximately 1200 Taka per person per month^(^ ^15^ ^)^) was 25 % or more. In each *upazila*, we obtained a list of all villages (the smallest administrative unit in Bangladesh, comprising approximately 250 households). Using a random number generator, each village was assigned a random number. Villages were sorted in ascending numerical order; the first fifty villages in this sorted list were assigned to the ‘Cash’ group, the next fifty villages were assigned to the ‘Food’ group, the next fifty villages were assigned to the ‘Cash & Food’ group, the next fifty villages were assigned to the ‘Cash + BCC’ group in the North and to the ‘Food + BCC’ group in the South, and the next fifty villages were assigned to the ‘Control’ group. In these selected villages, a census was carried out. From these data, a list of households was constructed that were considered poor (i.e. their predicted level of consumption – based on a score calculated using information on the age and education of the household head, housing characteristics, ownership of consumer durables, land ownership and household livelihoods – lies below the lower poverty line for rural Bangladesh); would have a child aged 0–24 months by the time the intervention began; and were not receiving benefits from any other social safety net interventions. These were the eligible households for participation in the study. Ten households meeting these three conditions were randomly selected from each village using simple random sampling, giving a total sample size of 2500 households targeted in each of the two studies.

### Study design and participants

The baseline survey was carried out in March–April 2012, prior to the first payment. The principal survey instrument was a multi-topic household questionnaire that included questions on maternal knowledge of infant and young child feeding (IYCF) and access to and use of micronutrients. Mothers of children under the age of 24 months at baseline (or if absent, the primary female caregivers, referred to hereafter as ‘mothers’) and their spouses provided answers to different sections based on who was most informed, with men interviewed by male enumerators and women interviewed by female enumerators. Mothers responded to all questions on micronutrient supplements. An endline survey was conducted in April 2014 during the final month of payments. Community questionnaires were administered to capture information on local infrastructure and access to services. CNW who implemented the nutrition training were also surveyed at endline.

### Measures

We considered five outcome measures. These are: (i) whether the mother could identify at least one adverse consequence of insufficient Fe intake by infants and young children; (ii) whether the mother was familiar with MMP; (iii) for each child aged 6–59 months, whether the mother had ever mixed MMP into the food the child consumed; (iv) for each child aged 6–59 months, whether the mother had at any time during the last 7d mixed MMP into the food the child consumed; and (v) for each child aged 6–59 months, whether the mother had at any time during the last 7d mixed MMP into the food the child consumed or provided an Fe supplement in some other form (tablets, syrup). Each outcome was set equal to 1 when the answer was yes and equal to 0 when the answer was no. Outcomes (i) and (ii) were collected at baseline and endline. For children aged 6–59 months, outcomes (iii), (iv) and (v) were collected at endline.

We used additional variables for attrition analysis and robustness checks, as well as for covariates in impact analysis. These included baseline maternal characteristics (age and grade of formal schooling), child characteristics (age in months and sex) and household characteristics (log value (in Taka) of production assets and consumer durables, household size, whether the household is female headed) from the household surveys; as well as locality characteristics (number of months during which local roads were impassable, the presence of individuals from non-TMRI organizations who also provided information on aspects of IYCF nutrition (called ‘IYCF promoters’)) from the community surveys.

### Sample size calculations

We calculated the *ex post* statistical power for the outcomes specific to the paper. Under the conservative assumption that baseline covariates included in analysis would provide no explanatory power for these outcomes, setting a significance level of 0·05 and statistical power of 0·80, and using outcome-specific means, standard deviations and intracluster correlations from the baseline data, we estimated that this sample provides sufficient statistical power to detect the following: a 10 percentage point increase in mothers who can identify one reason why Fe deficiency in children is a concern; an 8 percentage point increase in the likelihood that a mother has heard of MMP; a 10 percentage point increase in the likelihood that a child ever consumed MMP; a 5 percentage point increase in the likelihood that in the last 7d a child consumed MMP; and a 5 percentage point increase in the likelihood that in the last 7d a child consumed MMP, Fe tablets or Fe syrup. Actual power would differ if parameters changed between baseline and endline or if inclusions of baseline covariates led to power gains.

### Statistical analysis

Statistical analyses were conducted in the statistical software package STATA version 15.0. Household, maternal and child characteristics were compared across treatment and control arms separately for the North RCT and the South RCT. Using ANOVA, variables were considered balanced if *P*>0·05 for *F* tests comparing means for continuous variables and percentages for dichotomous variables across all treatment and control groups.

We assessed whether attrition was non-random by estimating a probit model where the dependent variable equalled 1 if the household attrited between baseline and endline, 0 otherwise. We included as covariates household treatment status, the baseline household and maternal characteristics used as controls for our impact estimates, baseline maternal knowledge of the consequences of Fe deficiency and baseline maternal awareness of MMP. Parameter estimates were converted to marginal effects^(^ ^16^ ^)^. A covariate had a statistically significant impact on the probability of a household attriting if *P*<0·05. Standard errors accounted for clustering at the village level^(^ ^16^ ^)^.

We conducted ANCOVA estimates for the impact of different treatment arms at endline on mothers’ knowledge of the consequences of insufficient Fe intake and on their familiarity with MMP controlling for baseline levels of maternal knowledge regarding the consequences of Fe deficiency, as well as other baseline maternal characteristics (age and grade of formal schooling) and household characteristics (log value (in Taka) of production assets and consumer durables; household size; whether the household is female headed). Single-difference impact estimates were used to measure the impact of different treatment arms on the child-level outcomes. These controlled for the same baseline maternal and household characteristics listed above as well as child age (in months) and sex. Because the outcomes are dichotomous variables, we used a probit estimator. Estimated coefficients were transformed into marginal effects; for our treatment variables which are dichotomous, these were obtained by calculating the predicted change in our dichotomous outcomes when we changed the value of the dummy variable for treatment from 0 to 1^(^ ^16^ ^)^. We report a pseudo *R* ^2^ statistic to assess goodness-of-fit^(^ ^16^ ^)^. Standard errors account for clustering at the village level^(^ ^16^ ^)^. Impacts were considered statistically significant if *P*<0·05. We used Wald *χ* ^2^ statistics to test for differences in impacts across treatment arms^(^ ^16^ ^)^.

Robustness checks included assessing whether the impact estimates were sensitive to the inclusion or exclusion of control variables, disaggregating the sample by maternal and child characteristics, disaggregating by locality characteristics such as market accessibility and the presence of non-TMRI IYCF promoters, and whether the results were sensitive to the use of alternative estimation methods (logits, linear probability models and linear probability models with union fixed effects (unions are the geographic unit above a village)).

## Results

### Programme implementation

Quantitative data collected throughout the intervention indicated that the TMRI transfers and BCC were implemented as designed. Women in the BCC treatment arms attended nearly all weekly sessions. Average attendance in the North among women in BCC treatment arms was forty-seven sessions per year and in the South forty-eight sessions per year – with each session lasting approximately an hour. When a session was missed, 83 % of respondents reported that the CNW followed up with a home visit. Further, CNW delivering the BCC messages were knowledgeable. The endline survey of CNW included a fourteen-question quiz on key nutrition messages they were supposed to provide to beneficiaries regarding exclusive breast-feeding; the introduction of complementary foods; the importance of diet diversification; micronutrients; and water, sanitation and health. The mean score out of 14 was 13·2 in the North and 13·5 in the South.

### Trial profile and attrition

In the North RCT, 2500 households were invited to participate in the study. There were two refusals, yielding a baseline sample of 2498 households (Fig. 1). At endline, across all arms we obtained complete data for 2408 households; these household contained 2698 children aged 6–59 months. The household attrition rate was 3·6 % with most of this arising from out-migration. Attrition probit models found that households in the ‘Cash & Food’ treatment group were 2·4 percentage points less likely to attrit relative to control households (*P*=0·02) and female-headed households were 3·1 percentage points more likely to attrit (*P*=0·03). No other variables were associated with attrition in the North.

In the South RCT, 2500 households were invited to participate in the study. There were six refusals, yielding a baseline sample of 2494 households (Fig. 2). At endline, across all arms we obtained complete data for 2432 households; these household contained 2775 children aged 6–59 months. The household attrition rate was 2·5 % with most of this arising from out-migration. In the South, analysis of attrition found no association between treatment variables, household or maternal characteristics and attrition. One community characteristic, months in which roads were passable, was associated with lower attrition. Each month that a village road was passable reduced the likelihood of attrition by 0·1 % (*P*=0·04).

In the North, across all treatment arms the sample is balanced across baseline household characteristics, baseline maternal characteristics (including knowledge of Fe deficiency in children and awareness of MMP), and endline child age and sex (Table 1). In the South, the sample is balanced across baseline household characteristics (except for land ownership), endline child characteristics, baseline maternal age and knowledge of Fe deficiency in children, but not for baseline maternal schooling or awareness of MMP (Table 1).

**Table 1:** Baseline characteristics of households, mothers and children by study and treatment arm, Bangladesh, March 2012–May 2014

	‘Cash’		‘Food’		‘Cash & Food’		‘Cash + BCC’		‘Food + BCC’		‘Control’		
	Mean	sd	Mean	sd	Mean	sd	Mean	sd	Mean	sd	Mean	sd	*P*
North RCT													
Log value of per capita household assets	6·84	1·04	6·91	1·03	6·75	0·98	6·88	1·02	–	–	6·88	1·06	0·11
Land owned (decimals*)	13·8	37·6	14·9	33·4	12·4	37·2	13·4	32·7	–	–	15·0	32·6	0·74
Household size (no. of persons)	4·9	1·5	4·9	1·5	4·9	1·45	4·9	1·5	–	–	4·9	1·4	0·98
Female head (%)	5·9	23·7	9·7	29·6	7·6	26·6	8·0	27·2	–	–	6·7	25·1	0·17
Mother’s age (years)	30·4	9·9	30·9	10·3	30·2	8·9	30·5	9·5	–	–	30·4	9·9	0·85
Mother’s schooling (grade)	2·3	3·0	2·2	2·9	2·1	2·8	2·1	2·9	–	–	2·4	3·0	0·49
Mother knows about Fe deficiency (%)	79·7	40·3	81·6	38·8	78·5	41·1	81·3	39·0	–	–	77·4	41·9	0·34
Mother heard of MMP (%)	24·1	42·8	27·9	48·9	28·5	45·2	24·4	43·0	–	–	25·1	43·4	0·30
Child’s age (months)	36·7	9·9	36·0	10·3	35·7	11·2	35·9	10·7	–	–	35·5	10·5	0·34
Child is female (%)	47·9	50·0	47·3	50·0	48·5	50·0	49·7	50·0	–	–	46·5	49·9	0·87
No. of mothers sampled	483		485		490		475		–		475		–
No. of children sampled	534		531		559		541		–		533		–
South RCT													
Log value of per capita household assets	7·13	1·06	7·20	1·08	7·30	1·10	–	–	7·21	1·11	7·23	1·11	0·23
Land owned (decimals*)	17·8	32·7	18·1	33·4	26·7	61·4	–	–	22·8	48·9	22·4	48·4	<0·01
Household size (no. of persons)	5·4	1·5	5·5	1·6	5·4	1·7	–	–	5·3	1·6	5·6	1·7	0·12
Female head (%)	8·9	28·5	12·4	33·0	10·8	31·1	–	–	12·3	32·9	12·1	32·6	0·28
Mother’s age (years)	31·3	9·8	32·0	10·6	32·4	11·6	–	–	31·1	10·3	31·5	10·5	0·26
Mother’s schooling (grade)	2·7	3·0	2·6	2·9	2·9	3·0	–	–	3·1	3·1	3·2	3·2	0·03
Mother knows about Fe deficiency (%)	60·9	48·8	60·3	49·0	61·0	48·8	–	–	59·2	49·2	62·4	48·5	0·87
Mother heard of MMP (%)	17·1	37·7	13·4	31·1	16·6	37·3	–	–	13·0	33·6	19·2	39·4	0·02
Child’s age (months)	35·9	10·4	34·4	10·9	35·4	11·2	–	–	34·7	11·7	35·7	10·5	0·11
Child is female (%)	53·4	49·9	45·6	49·8	48·3	50·0	–	–	46·2	49·9	48·4	50·0	0·08
No. of mothers sampled	490		489		482		–		487		484		–
No. of children sampled	539		566		551		–		563		556		–

BCC, (high-quality nutrition) behaviour change communication; RCT, randomized controlled trial; MMP, multiple-micronutrient powders.

* A decimal is 0·1 ha.

### Impacts on maternal knowledge of iron deficiency and multiple-micronutrient powders

In the North, mothers’ knowledge of the adverse consequences of Fe deficiency increased by 11·9 percentage points in the ‘Cash + BCC’ treatment arm (*P*≤0·01) relative to the control group (Table 2). It increased by 6·2 percentage points in the ‘Cash & Food ‘treatment arm (*P*=0·04). There was no statistically significant change in any other treatment arm. Awareness of MMP was 29·0 percentage points higher in the ‘Cash + BCC’ treatment arm (*P*≤0·01) relative to the control group. It was 11·4 percentage points higher in the ‘Cash’ treatment arm (*P*=0·02) relative to the control group. Awareness of MMP was 17·5 percentage points higher in the ‘Cash + BCC’ treatment arm compared with the ‘Cash’ treatment arm, with this difference being statistically significant (*P*≤0·01).

**Table 2:** Impact estimates of treatment arms on maternal knowledge by study and treatment arm, Bangladesh, March 2012–May 2014

	Mother can identify one reason why Fe deficiency in children is a concern			Mother has heard of MMP		
	Marginal effect	se	*P*	Marginal effect	se	*P*
North RCT						
‘Cash’	0·039	0·029	0·12	0·114	0·046	0·02
‘Food’	0·013	0·029	0·67	0·035	0·045	0·45
‘Cash & Food’	0·062	0·028	0·04	0·056	0·045	0·22
‘Cash + BCC’	0·119	0·024	<0·01	0·290	0·036	<0·01
Pseudo *R* ^2^	0·019			0·051		
South RCT						
‘Cash’	0·003	0·025	0·89	0·007	0·041	0·87
‘Food’	0·034	0·023	0·16	−0·013	0·039	0·72
‘Cash & Food’	−0·002	0·028	0·94	−0·008	0·041	0·85
‘Food + BCC’	0·092	0·024	<0·01	0·222	0·044	<0·01
Pseudo *R* ^2^	0·001			0·028		

MMP, multiple-micronutrient powders; RCT, randomized controlled trial; BCC, (high-quality nutrition) behaviour change communication. Marginal effects of probit models reported. ANCOVA estimates at endline control for baseline levels of maternal knowledge regarding the consequences of Fe deficiency, as well as other baseline maternal characteristics (age and grade of formal schooling) and household characteristics (log value (in Taka) of production assets and consumer durables; household size; whether the household is female headed). Standard errors account for clustering at the village level.

In the South, knowledge of the adverse consequences of Fe deficiency increased by 9·2 percentage points for mothers in the ‘Food + BCC’ treatment arm (*P*≤0·01) relative to the control group (Table 2). There was no statistically significant change in any other treatment arm. Awareness of MMP was 22·2 percentage points higher in the ‘Food + BCC’ treatment arm (*P*≤0·01) relative to the control group. For the ‘Food’ treatment arm, there was no statistically significant impact of knowledge of MMP (*P*=0·72). Awareness of MMP was 22·1 percentage points higher in the ‘Food + BCC’ treatment arm compared with the ‘Food’ treatment arm with this difference being statistically significant (*P*≤0·01).

In each of the North and the South, results for both knowledge of adverse consequences of Fe deficiency and awareness of MMP are robust to the inclusion or exclusion of maternal and household characteristics. Impact estimates on each treatment arm remained virtually unchanged when we estimated using a linear probability model or a union-level linear probability fixed-effects regression. In each region, we disaggregated the sample by maternal age, maternal education, household assets, the number of months during which local roads were impassable and the presence of a non-TMRI IYCF promoter. We found no statistically significant differences in these impacts across the different sub-samples.

### Impacts on child consumption of multiple-micronutrient powders and other iron supplements

MMP or other Fe supplements were available in 88 % of villages in the North and 78 % of villages in the South. In the North, children aged 6–59 months in the ‘Cash’ treatment arm were, at endline, 12·6 percentage points more likely to have ever consumed MMP (*P*=0·01) relative to the control group (Table 3). Children in the ‘Cash + BCC’ treatment arm were 32·0 percentage points more likely to have ever consumed MMP (*P*≤0·01) relative to the control group. Children in the ‘Cash’ and the ‘Cash + BCC’ treatment arms were more likely to have consumed MMP in the last 7d relative to the control group by 5·2 and 16·9 percentage points, respectively (both *P*≤0·01). All treatment arms had, relative to the control group, a statistically significant increase in the likelihood that children consumed MMP, Fe tablets or Fe syrup in last week with the magnitude of the impact estimates ranging from 7·0 percentage points (‘Food’) to 22·3 percentage points (‘Cash + BCC’). For each of these three outcomes, we tested the null hypothesis that the impacts of the ‘Cash’ and the ‘Cash + BCC’ treatment arms were equal; in each case we did not accept this null hypothesis (*P*≤0·01).

**Table 3:** Impact estimates of treatment arms on children’s consumption of multiple-micronutrient powders (MMP) and other iron supplements by study and treatment arm, Bangladesh, March 2012–May 2014

	Child has ever consumed MMP			Child has consumed MMP in the last 7d			Child has consumed MMP, Fe tablets or Fe syrup in the last 7d		
	Marginal effect	se	*P*	Marginal effect	se	*P*	Marginal effect	se	*P*
North RCT									
‘Cash’	0·126	0·048	0·01	0·052	0·024	0·01	0·090	0·033	<0·01
‘Food’	0·033	0·049	0·50	0·026	0·019	0·12	0·070	0·027	<0·01
‘Cash & Food’	0·036	0·047	0·44	0·037	0·024	0·06	0·080	0·031	<0·01
‘Cash + BCC’	0·320	0·044	<0·01	0·169	0·033	<0·01	0·223	0·039	<0·01
Pseudo *R* ^2^	0·055			0·107			0·073		
South RCT									
‘Cash’	0·014	0·032	0·65	0·000	0·009	0·98	0·028	0·017	0·07
‘Food’	0·039	0·034	0·24	0·000	0·009	0·98	0·005	0·015	0·75
‘Cash & Food’	0·021	0·033	0·51	0·001	0·008	0·92	0·027	0·018	0·10
‘Food + BCC’	0·119	0·037	<0·01	0·039	0·015	<0·01	0·071	0·022	<0·01
Pseudo *R* ^2^	0·037			0·070			0·033		

RCT, randomized controlled trial; BCC, (high-quality nutrition) behaviour change communication. Marginal effects of probit models reported. Single-difference estimates at endline control for baseline levels of maternal knowledge regarding the consequences of Fe deficiency, other baseline maternal characteristics (age and grade of formal schooling), household characteristics (log value (in Taka) of production assets and consumer durables; household size; whether the household is female headed) and child characteristics (age in months and sex). Standard errors account for clustering at the village level.

In the South, children aged 6–59 months in the ‘Food + BCC’ treatment arm were, at endline, 11·9 percentage points more likely to have ever consumed MMP (*P*≤0·01) relative to the control group and were 3·9 percentage points more likely to have consumed MMP in the last 7d (*P*≤0·01; Table 3). For these outcomes, no other treatment arm in the South had statistically significant impacts. Relative to the control group, ‘Food + BCC’ (7·1 percentage points) had a statistically significant effect on the likelihood that children consumed MMP, Fe tablets or Fe syrup in the last week (*P*≤0·01). We rejected the null hypotheses that the impacts of ‘Food’ and ‘Food + BCC’ on children ever consuming MMP, on consumption of MMP in the last 7d and on consumption of MMP, Fe tablets or Fe syrup were equal (*P*≤0·01) in all cases.

In both the North and the South, we disaggregated the child samples by child sex and age, by maternal education, by maternal age and by household wealth. In all cases, we did not reject the null hypothesis that the impact of ‘Cash + BCC’ (in the North) or ‘Food + BCC’ (in the South) did not differ across these disaggregations. We also disaggregated by the number of months during which local roads were impassable and the presence of a non-TMRI IYCF promoter, and found no statistically significant differences in these impacts across these different sub-samples in either the North or the South.

## Discussion

We found that a transfer (food or cash) accompanied by high-quality nutrition BCC improved mothers’ average knowledge of Fe deficiency and awareness of MMP, as well as significantly increased the likelihood of their children aged 6–59 months consuming MMP or some other Fe supplement (tablets, syrup) in the preceding week. The BCC drove these effects: in all cases, improvements were statistically significant relative to not only the control group, but also relative to the group that received the corresponding transfer only (‘Cash’ in the North; ‘Food’ in the South). In the North, receiving a cash transfer alone also significantly increased mothers’ awareness of MMP, as well as children’s likelihood of consuming MMP ever or in the preceding week. It is possible that the receipt of cash resulted in mothers frequenting markets or health centres where these supplements were sold, and this exposure resulted in improved awareness and use of MMP. However, no similar effect was observed in the South and, as noted above, in the North the impact of receiving cash was smaller than that of receiving both cash and nutrition BCC.

Our study has strengths. Implementation of the interventions was of high quality and as designed. Our analyses were based on longitudinal data with rich information on over 5000 children, their mothers, their households and their localities. Attrition was low. Our findings were robust to changes in estimation approach and disaggregation.

Our study also has weaknesses. Given the design of the RCT, we cannot directly compare impacts across the North and the South. Neither RCT included a ‘BCC only’ arm. For two outcomes (mother can identify one reason why Fe deficiency in children is a concern; mother has heard of MMP), we have baseline and endline data, but for the other three (child has ever consumed MMP; child has consumed MMP in the last 7d; child has consumed MMP, Fe tablets or Fe syrup in the last 7d), we only have endline data. We did not measure anaemia status. Attrition in the North was significantly associated with being in the ‘Food & Cash’ arm; however, the magnitude is small (2·4 percentage points less likely to attrit relative to control households) and given the pattern of impacts, does not appear to drive the results. Some household and maternal characteristics showed statistically significant differences at baseline in the South; however, magnitudes of difference were small, and these characteristics along with others were included as control variables in impact estimation.

Existing evidence suggests that free provision of MMP is effective in reducing IDA among children of pre-school age but leaves knowledge gaps on alternative delivery platforms with greater sustainability and scalability^(^ ^9^ ^,^ ^17^ ^,^ ^18^ ^)^. The one study of a market-based approach that we are aware of, in which MMP were sold by front-line health workers in Bangladesh, found that household poverty and variable household awareness of MMP were important constraints to effectiveness^(^ ^19^ ^)^. Moreover, increasing awareness through counselling on IYCF was also insufficient to meaningfully increase uptake^(^ ^19^ ^)^. Our study complements this work, by examining in two RCT how increasing household income, with or without also increasing household awareness of MMP and other Fe supplements, affects households’ purchase and use of the supplements, in a setting where they are widely available in the market. We find that simply increasing income of mothers in localities where MMP were widely available through either cash or in-kind transfers had no or at best small effects on increasing the use of MMP or related supplements. However, combining transfers with intensive high-quality BCC that increased mothers’ knowledge of Fe deficiency and awareness of supplements led to significant increases in the likelihood that their children aged 6–59 months had ever consumed MMP (32 and 11·9 percentage points, *P*≤0·01), consumed MMP in the preceding week (16·9 and 3·9 percentage points, *P*≤0·01) and consumed either MMP or an Fe supplement in the preceding week (22·3 and 7·1 percentage points, *P*≤0·01). These results suggest that relaxing only the income constraint of poor households will not in general be sufficient to increase uptake of MMP and related supplements. However, relaxing both the income and awareness constraints together may be effective in a setting where the supplements are widely available. Taken together with the previous study in which relaxing only the awareness constraint appeared to be inadequate, our findings suggest that effective, scalable platforms may need to twin interventions that increase income with interventions that increase awareness of MMP and other supplements.

The results of our study build the evidence base for social protection programming as a promising platform to increase the number of children, particularly children in poor households, who could be reached with MMP. These findings provide proof of concept that nutrition-sensitive interventions through social protection – and particularly transfers with BCC added – can be a promising way to advance progress on micronutrient deficiencies such as IDA, allowing a scale beyond what may be feasible or sustainable for nutrition-specific interventions such as free distribution of MMP.
